# Editorial: Brain-computer interfaces in neurological disorders: expanding horizons for diagnosis, treatment, and rehabilitation

**DOI:** 10.3389/fnins.2024.1526723

**Published:** 2024-11-29

**Authors:** Nan Wang, Wen-Jun Tu

**Affiliations:** Department of Neurosurgery, Beijing Tiantan Hospital, Capital Medical University, Beijing, China

**Keywords:** brain-computer interface, neurological, rehabilitation, stroke, spinal cord injury

Brain-computer interfaces (BCIs) are at the forefront of medical technology, with transformative potential for neurological rehabilitation (Dobkin, 2007). Motor imagery-based brain-computer interfaces (MI-BCIs) hold significant clinical value in neurorehabilitation (Liu et al., 2023; Omari et al., 2024). By facilitating direct communication between the brain and external devices, BCIs are broadening the scope of neurorehabilitation, particularly for individuals with conditions such as spinal cord injury (SCI), stroke, and unilateral spatial neglect (USN) (Daly and Wolpaw, 2008; Yang et al., 2021; Fu et al., 2022). This Research Topic aims to highlight recent advancements, diverse applications, and the complex challenges associated with BCIs in managing neurological disorders. It includes four notable contributions, each providing unique insights into the potential of BCIs to enhance quality of life and functional abilities for those affected.

In the study by Guo et al. on spinal cord injury (SCI), the authors explored a novel approach combining epidural electrical stimulation (EES) with near-infrared nerve stimulation (nINS) to enhance motor function specificity in SCI rats. The results revealed that adding optical stimulation to conventional EES could selectively activate target muscles with reduced stimulation intensity, offering an effective strategy to mitigate unwanted muscle activation and improve movement control. This dual-stimulation approach presents a promising new avenue for addressing motor dysfunctions associated with SCI, paving the way for more precise neuromodulation in rehabilitation settings (Guo et al.).

Feitosa et al. examined the efficacy of virtual reality (VR)-based rehabilitation in stroke patients. Using graph theory, the study assessed cortical reorganization in individuals undergoing VR-assisted therapy. The results demonstrated that the VR-based rehabilitation tool, Gesture Collection, significantly enhanced functional connectivity within motor-related brain regions compared to standard therapy. The improvement in connectivity, particularly within the frontoparietal and somatosensory networks, highlights the potential of VR to promote neuroplasticity and facilitate motor recovery in stroke survivors. By providing engaging and targeted motor exercises, VR-assisted rehabilitation could serve as a valuable complement to traditional stroke recovery programs (Feitosa et al.).

Wang et al. investigated the neural characteristics associated with neuropathic pain and numbness in SCI patients. Using electroencephalography (EEG), the study identified distinct brain network patterns differentiating patients with neuropathic pain from those experiencing numbness. Notably, individuals suffering from pain exhibited reduced power in lower frequency bands (θ and α) and increased power in the higher frequency band (β), accompanied by altered network connectivity. These findings suggest that EEG-based metrics could serve as valuable biomarkers for distinguishing different neuropathic symptoms in SCI patients, thereby informing personalized intervention strategies to improve chronic pain management in this population (Wang et al.).

The article by Gouret et al. provides a focused review of brain-computer interface (BCI) applications in the context of unilateral spatial neglect (USN), a common yet often underrecognized consequence of stroke. The review emphasizes the limited use of BCIs in addressing cognitive deficits, particularly visuo-attentional impairments, and underscores the need for expanded research in this area. The authors propose integrating VR with BCIs as a rehabilitation tool for USN, leveraging the cognitive engagement provided by VR to support attentional recovery. This perspective advocates for broadening the scope of BCI applications beyond motor rehabilitation to include cognitive therapy, thereby addressing a significant gap in current neurological treatment approaches (Gouret et al.).

Collectively, these studies highlight the versatility and potential of brain-computer interfaces (BCIs) in neurological rehabilitation. By enhancing motor recovery, managing pain, and supporting cognitive restoration, BCIs represent a multidimensional tool capable of addressing various aspects of neurological impairments (He et al., 2024). However, several challenges remain, including the need for improved user accessibility, device usability, and ethical considerations. As BCI technology continues to advance, interdisciplinary collaboration will be crucial for translating experimental insights into real-world solutions that can be widely adopted in clinical practice. For instance, integrating optogenetic BCIs with functional imaging techniques could allow targeted modulation of specific circuits, further promoting neurorehabilitation (Tang et al., 2024; Chai et al., 2024).

This Research Topic aims to inspire ongoing research and innovation in the field of BCIs, with the ultimate goal of empowering individuals with neurological disorders to lead more independent and fulfilling lives. Through continued exploration and application, BCIs hold the promise of bridging the gap between neurological impairment and functional recovery, offering hope to patients, clinicians, and researchers alike.
